# Socioeconomic status and metabolic syndrome in the general population of China: a cross-sectional study

**DOI:** 10.1186/1471-2458-12-921

**Published:** 2012-10-30

**Authors:** Yiqiang Zhan, Jinming Yu, Ruoqing Chen, Junling Gao, Rongjing Ding, Yuanyuan Fu, Lijun Zhang, Dayi Hu

**Affiliations:** 1 Institute of Clinical Epidemiology, Key Laboratory of Public Health Safety (Ministry of Education), Fudan University, Shanghai, 200032, PR, China; 2 Heart Center, Peking University People’s Hospital, Beijing, 100044, PR, China

**Keywords:** Socioeconomic status, Metabolic syndrome, Cross-sectional study

## Abstract

**Background:**

Individual socioeconomic status (SES) has been found to be associated with cardiovascular diseases in developed countries, but the association between individual SES and metabolic syndrome (MetS) is still unclear in China. The objective of this study was to investigate the association between individual SES and MetS in China.

**Methods:**

A cross-sectional study of 10054 community residents was performed from May to August 2007 using multistage stratified random sampling. SES was assessed in terms of education, personal monthly income, and household monthly income. The association between SES and MetS was determined by logistic regression models.

**Results:**

After the adjustments regarding age, marital status, smoking, drinking, physical activity, body mass index (BMI), and community type, odds ratios (ORs) for MetS of individuals with education level of 7~12 years and >12 years were 0.87 (95% confidence interval [CI]: 0.75 to 0.99) and 0.83 (95% CI: 0.62 to 0.91) respectively compared with those with education level of <7 years in women. Following the adjustments as above, ORs for MetS of individuals with household monthly income level of middle and higher were 0.94 (95% CI: 0.86 to 0.97), and 0.72 (95% CI: 0.65 to 0.88) respectively compared with those with lower household monthly income level in women. The association between SES and MetS was not significant in men.

**Conclusions:**

Gender had an influence on the association between individual SES and MetS. Lower education and household monthly income level were associated with higher risk of MetS among community residents in women, while such association was not significant in men.

## Background

Previous studies showed that lower socioeconomic status (SES) was associated with higher risk of cardiovascular disease (CVD) in developed countries [1-4]. Distribution of risk factors of CVD among different SES strata may be the potential explanations. However, the association between SES and metabolic syndrome (MetS) has been rarely investigated both in developed and developing countries [5-7]. In China, most of the researchers studying risk factors of MetS have attached importance to biochemistry and anthropometric indicators which are micro-level determinants of health [8,9], while the studies of social determinants of health such as SES are still scarce [10].

Levels of education and income, the indicators of SES measurement, may have effects on the prevalence of MetS. In South Korea, lower education level and personal income were associated with a higher risk of the MetS in women [5]. Moreover, in developed countries, individuals with lower occupation level had a higher risk of MetS compared with those of higher occupation level [11]. Even in Jamaica, where prevalence of MetS was low, central obesity, one component of MetS was reversely related to occupation and education in women [12]. Likewise, in North India, higher SES and sedentary occupation were found to be associated with MetS [13]. All of these studies suggested that SES may affect MetS, especially in China which was facing great social and economic transition in the last decade.

It was reported that the prevalence of MetS across China was 9.8% and 17.8% for men and women respectively in 2000 [14]. But in Jiangsu Province, the prevalence of MetS was as high as 31.5% in 2002 [10], and recent studies showed a rapidly increasing prevalence of MetS in China [10,14-17]. Beijing is a highly urbanized city with large income, education, and health inequality, but there seems to be few studies regarding SES and MetS from this region. The present study was conducted to investigate the association between individual SES inequality and MetS among adults using data from a cross-sectional health survey in Beijing.

## Methods

### Design and study population

All participants were provided written informed consent, and the ethical approval was obtained from the Ethic Committee of Beijing Municipal Science and Technology Commission. This study was a cross-sectional chronic diseases and risk factors survey conducted in Beijing from May 2007 to August 2007. Citizens or permanent residents (those who are registered Beijing citizens excluding those who lived outside of Beijing for no less than six months and non-registered Beijing citizens who have temporary residence permit and have lived in Beijing for no less than six months) who were 18 or older were recruited using multistage stratified random sampling design. We select the first sampling unit according to the per capita GDP (gross domestic product) of each district followed by the second sampling unit (towns and urban neighborhoods). Then urban neighborhood communities and villages are selected as the final sampling unit. The final study population were recruited from the final sampling unit (communities). Finally, Two urban administrative districts, one urban–rural mixed district, and one rural district were selected, and then 38 communities (17 urban communities and 21 rural communities) were randomly sampled. Mainland China has a unique residence registration system, through which we could obtain the basic demographic information, such as name, birth date, and gender stored in the departments of local governments. Before the survey, we informed local administrators of the aim and method of our study. Due to their help, we could disseminate our study design via broadcasting and booklets. All of the permanent families in the communities were informed to participate in our survey and only one member in a family was invited for the survey due to the consideration of genetic predisposition. Inclusion criteria included that Beijing citizens or permanent residents who were 18 years or older and provided written informed consents. Exclusion criteria included that those who were pregnant or disabled for physical examinations and interviews, or did not provide written informed consents, or dropped out after recruitment. And at the night before our survey, the residents were told not to drink or eat from 8 pm to 8 am the day after that.

### Health interview

The health interview was performed by trained medical staff at community clinics using a well-established questionnaire to determine demographic, socioeconomic, and behavioral characteristics of the study population. Demographic information included birthday, gender, race, marital status, and community name. Socioeconomic status included education, personal monthly income, and household monthly income. Behavioral information included smoking status, drinking status, and physical activities. Marital status was classified as married, unmarried, or else (divorced or widowed). Education level was categorized as elementary school or lower (<7 years), middle or high school (7~12 years), and college or higher (>12 years). Personal monthly income was divided into lower (<RMB [Chinese yuan] 2000), middle (RMB 2000~4000) and higher (>RMB 4000). Household monthly income per person was divided into lower (<RMB 1000), middle (RMB 1000~2000), and higher (>RMB 2000), on the basis of the estimation of personal monthly income in Beijing—the average personal monthly income in 2006 being RMB 3000. Participants were also divided into current smokers, ex-smokers, and non-smokers. Drinking status was classified as always, sometimes, and never. Physical activity was categorized as ≤1 hour/day, 2–3 hours/day, and ≥4 hours/day.

### Physical examination

Physical examination included anthropometric measurement, blood pressure measurement, medical history, and drug administration history. Height and weight were measured to the nearest 0.1 cm and 0.1 kg respectively with the subject standing barefoot in light clothes. Waist circumference was measured to the nearest 0.1 cm at the mid-point between the 12th rib and right anterior superior iliac spine. Body mass index (BMI) was calculated as weight (kg) to be divided by square of height (m). Blood pressure was measured using standard mercury sphygmomanometer on the right arm in sitting position after the participants rested for 5 minutes. Phase 1 and phase 5 Korotkoff sound was used as systolic blood pressure (SBP) and diastolic blood pressure (DBP) respectively. Blood pressure was measured twice with the average results for the data analysis. Medical history and drug administration history were obtained from medical record and confirmed by community physicians. All the measurements were adopted by community licensed physicians.

### Blood sample test

Blood samples were collected from all the participants after an overnight fasting. All the biochemical measurements were conducted in the central laboratory of Peking University People’s Hospital. Concentrations of fasting glucose, total cholesterol (TC), high-density lipoprotein cholesterol (HDL-C), triglycerides (TG), and low-density lipoprotein cholesterol (LDL-C) were measured using an auto analyzer (Hitachi 717, Hitachi Instruments Inc., Tokyo, Japan).

### Determination of SES and MetS

As mentioned above, SES was measured in terms of education level, personal monthly income, and household monthly income per person. All the information about SES was self-reported. The MetS was defined according to the *National Cholesterol Education Program* (NCEP) *Expert Panel on Detection, Evaluation, and Treatment of High Blood Cholesterol in Adults* (ATP III) [18]. Subjects meeting three or more of the following criteria were classified as MetS: waist circumference ≥102 cm in men or ≥ 88 cm in women; serum triglyceride (TG) concentration ≥150 mg/dL; HDL-C concentration ≤40 mg/dL in men or ≤ 50 mg/dL in women; blood pressure ≥130/85 mmHg; and serum glucose concentration ≥110 mg/dL. Those with a history of hypertension, diabetes mellitus, or hyperglycemia were considered to match the relative criteria. Subjects who were using medications for hypertension, diabetes, higher TG, or lower HDL met the criteria for high blood pressure, high fasting glucose, higher TG, and lower HDL respectively.

### Statistical analysis

All the statistics were obtained using SAS 9.2. Continuous variables were presented as mean ± standard deviation (SD) and categorical variables were presented as frequency and proportion. Cochran-Armitage was used for trend test across SES. The association between SES and MetS was analyzed using unconditional logistic regression models. Independent variables included age, gender, marital status, smoking, drinking, physical activities, and SES. Communities were categorized as urban, urban–rural mixed, and rural. Before analysis we examined the interaction effects of gender and SES indicators, and all of the interaction terms were statistically significant (*P*<0.01). The first logistic regression model included no other covariates. While the second logistic regression model was adjusted for age, the third model was adjusted for age, marital status, smoking, drinking, physical activities, and community type. ORs with 95% *CI* were also presented. All the analyses were two-tailed and *P*<0.05 was considered to be statistically significant.

## Results

### Basic characteristics of the study population

Table 1 showed the basic demographic statistics of the study population. Of the 10054 individuals, the prevalence of MetS for men and women was 14.05% and 28.55% respectively.

**Table 1:** Demographic characteristics of the study population (n=10054)

**Variable**	**Men (n=3 687)**	**Women (n=6 367)**	***P *value**
Age (years)	52.7±13.57	52.46±13.03	0.0053
BMI (kg/m^2^)	25.0±3.66	25.5±4.08	<0.0001
Waist circumference (cm)	89.17±10.36	85.5±10.80	0.0046
SBP (mmHg)	129.11±18.47	126.03±19.68	<0.0001
DBP (mmHg)	81.88±10.72	78.97±10.27	0.0030
FBG (mmol/L)	5.08±1.69	5.11±1.70	0.7639
TG (mmol/L)	1.63±1.73	1.46±1.24	<0.0001
HDL (mmol/L)	1.26±0.38	1.36±0.29	<0.0001
Education level			<0.0001
<7 years	810 (21.97)	2114 (33.20)	
7~12 years	2389 (64.80)	3635 (57.09)	
>12 years	488 (13.24)	618 (9.71)	
Personal monthly income level			<0.0001
Lower	3062 (83.05)	5841 (91.74)	
Middle	511 (13.86)	474 (7.44)	
Higher	114 (3.09)	52 (0.82)	
Household monthly income level			0.8124
Lower	3217 (87.25)	5535 (86.93)	
Middle	427 (11.58)	762 (11.97)	
Higher	43 (1.17)	70 (1.10)	
Marital status			<0.0001
Married	3384 (91.78)	5639 (88.57)	
Else	303 (8.22)	728 (11.43)	
Drinking			<0.0001
Never	1824 (49.47)	6061 (95.21)	
Previous drinking	561 (15.22)	181 (2.84)	
Current drinking	1302 (35.31)	124 (1.95)	
Smoking			<0.0001
Non-smoker	987 (26.77)	5780 (90.78)	
Current smoker	2191 (59.43)	483 (7.59)	
Ex-smoker	509 (13.81)	104 (1.63)	
Physical activity (hours/day)			0.0007
≤1	2482 (67.32)	4056 (63.70)	
2~3	1083 (29.37)	2102 (33.01)	
≥4	122 (3.31)	209 (3.28)	
Community type			<0.0001
Urban	663 (17.98)	1396 (21.93)	
Urban–rural mixed	1039 (28.18)	1999 (31.40)	
Rural	1985 (53.84)	2972 (46.68)	
Metabolic syndrome			<0.0001
Yes	518 (14.05)	1818 (28.55)	
No	3169 (85.95)	4549 (71.45)	

*BMI* body mass index, *SBP* systolic blood pressure, *DBP* diastolic blood pressure, *FBG* fasting blood glucose, *TG* triglycerides, *HDL* high density lipoprotein.

### Prevalence of MetS and its components by SES in Men and Women

Generally, as was shown in Table 2, the prevalence of MetS showed an inverse association between each indicator of SES in women, which was the same as that of abdominal obesity, high blood pressure, and high blood glucose. However, this trend was only found in terms of high blood pressure in men, as was shown in Table 2. More specifically, as for women in Table 2, the prevalence of high TG and low HDL decreased significantly only with increasing education level. And no such trend was shown between that of personal monthly income level or household monthly income level. Regarding men in Table 2, the prevalence of high TG increased significantly from lower SES indicators to higher SES indicators, while that of low HDL was only found significantly in terms of education level and personal monthly income level.

**Table 2:** Prevalence of metabolic syndrome and its component by socioeconomic status in women and men

**Variables**	**Metabolic syndrome**	**Abdominal obesity**	**High triglycerides**	**Low HDL-cholesterol**	**High blood pressure**	**High blood glucose**
Women						
Education level						
<7 years	842 (39.85)	1120 (53.01)	667 (31.57)	979 (46.33)	1390 (65.78)	453 (21.44)
7~12 years	869 (23.91)	1156 (31.80)	899 (24.73)	1607 (44.21)	1626 (44.73)	407 (11.20)
>12 years	107 (17.31)**	109 (17.64)**	160 (25.89)**	246 (39.81)**	202 (32.69)**	43 (6.96)**
Personal monthly income level						
Lower	1708 (29.25)	2281 (39.06)	1583 (27.11)	2606 (44.62)	3018 (51.68)	848 (14.52)
Middle	103 (21.73)	95 (20.04)	128 (27.00)	202 (42.62)	181 (38.19)	50 (10.55)
Higher	7 (13.46)**	9 (17.31)**	15 (28.85)	24 (46.15)	19 (36.54)**	5 (9.62)*
Household monthly income level						
Lower	1621 (29.29)	2175 (39.30)	1497 (27.05)	2473 (44.69)	2863 (51.73)	807 (14.58)
Middle	185 (24.28)	195 (25.59)	210 (27.56)	327 (42.91)	329 (43.18)	90 (11.81)
Higher	12 (17.14)**	15 (21.43)**	19 (27.14)	32 (45.71)	26 (37.14)**	6 (8.57)*
Men						
Education level						
<7 years	105 (12.96)	83 (10.25)	152 (18.77)	128 (15.80)	528 (65.19)	137 (16.91)
7~12 years	349 (14.61)	223 (9.33)	744 (31.14)	442 (18.50)	1320 (55.25)	334 (13.98)
>12 years	64 (13.11)	36 (7.38)	180 (36.89)**	113 (23.16)**	240 (49.18)**	69 (14.14)
Personal monthly income level						
Lower	418 (13.65)	283 (9.24)	827 (27.01)	541 (17.67)	1763 (57.58)	445 (14.53)
Middle	81 (15.85)	47 (9.20)	196 (38.36)	117 (22.90)	279 (54.60)	82 (16.05)
Higher	19 (16.67)	12 (10.53)	53 (46.49)**	25 (21.93)**	46 (40.35)**	13 (11.40)
Household monthly income level						
Lower	452 (14.05)	309 (9.61)	898 (27.91)	584 (18.15)	1861 (57.85)	465 (14.45)
Middle	59 (13.82)	28 (6.56)	159 (37.24)	90 (21.08)	210 (49.18)	71 (16.63)
Higher	7 (16.28)	5 (11.63)	19 (44.19)**	9 (20.93)	17 (39.53)**	4 (9.30)

*P<0.05 from Cochran-Armitage test for trend.

**P<0.01 from Cochran-Armitage test for trend.

### Smoking and drinking status by socioeconomic status in Men and Women

As was shown in Table 3, the proportion of those who were currently smoking and drinking decreased with increasing SES levels in men. This trend also presented in women for current smoking. But it was not statistically significant in women for current drinking.

**Table 3:** Smoking and drinking status by socioeconomic status in women and men

**Variables**	**Women**		**Men**	
	**Current smoking**	**Current drinking**	**Current smoking**	**Current drinking**
Education level				
<7 years	276 (13.06)	51 (2.41)	454 (56.05)	306 (37.78)
7~12 years	191 (5.25)	63 (1.73)	1527 (63.92)	859 (37.59)
>12 years	16 (2.59)**	10 (1.62)	210 (43.03)**	142 (20.08)**
Personal monthly income level				
Lower	466 (7.98)	117 (2.00)	1880 (61.40)	1139 (37.20)
Middle	11 (2.32)	7 (1.48)	257 (50.29)	136 (26.61)
Higher	6 (11.54)**	0 (0.00)	54 (47.37)**	27 (23.68)**
Household monthly income level				
Lower	449 (8.11)	115 (2.08)	1973 (61.33)	1189 (36.96)
Middle	33 (4.33)	9 (1.18)	193 (45.20)	99 (23.19)
Higher	1 (1.43)**	0 (0.00)	25 (58.14)**	14 (32.56)**

*P<0.05 from Cochran-Armitage test for trend.

**P<0.01 from Cochran-Armitage test for trend.

### Odds ratios for MetS based on SES in Men and Women

Table 4 showed the ORs and the corresponding 95% *CI* for the association between SES levels and MetS using logistic regression models for women and men respectively. Model 1 showed estimates of association between each indicator of SES and MetS without the adjustments. Model 2 was adjusted for age as a confounder. In Model 3, traditional risk factors, such as marital status, smoking, drinking, BMI, and physical activity were adjusted as potential mediators. Age and community type were also included in Model 3 as confounders. As for men, the associations between SES levels and MetS were not statistically significant in all the three models, while when regarding women, those associations between education level and household monthly income level and MetS were still significant even after adjustments for other covariates.

**Table 4:** Associations for the socioeconomic status and metabolic syndrome in women and men

**Variables**	**Models**		
	**OR (95% CI)**	OR (95% CI)*	OR (95% CI)**
Women			
Education level			
<7 years	1.0	1.0	1.0
7~12 years	0.43 (0.38,0.49)	0.80 (0.69,0.92)	0.87 (0.75,0.99)
>12 years	0.26 (0.20,0.33)	0.57 (0.44,0.73)	0.83 (0.62,0.91)
Personal monthly income level			
Lower	1.0	1.0	1.0
Middle	0.64 (0.50,0.80)	0.87 (0.68,1.11)	0.98 (0.93,1.59)
Higher	0.36 (0.16,0.80)	0.48 (0.21,1.13)	0.56 (0.22,1.39)
Household monthly income level			
Lower	1.0	1.0	1.0
Middle	0.72 (0.60,0.87)	0.86 (0.71,0.94)	0.94 (0.86,0.97)
Higher	0.45 (0.24,0.85)	0.65 (0.34,0.95)	0.72 (0.65,0.88)
Men			
Education level			
<7 years	1.0	1.0	1.0
7~12 years	1.12 (0.88,1.42)	1.18 (0.91,1.54)	1.06 (0.79,1.42)
>12 years	0.89 (0.62,1.26)	0.96 (0.66,1.39)	0.93 (0.61,1.44)
Personal monthly income level			
Lower	1.0	1.0	1.0
Middle	1.11 (0.85,1.45)	1.12 (0.86,1.47)	0.98 (0.72,1.33)
Higher	1.18 (0.70,1.98)	1.22 (0.72,2.05)	1.18 (0.66,2.12)
Household monthly income level			
Lower	1.0	1.0	1.0
Middle	0.90 (0.66,1.22)	0.91 (0.66,1.24)	0.83 (0.59,1.19)
Higher	1.10 (0.48,2.50)	1.12 (0.49,2.57)	0.94 (0.38,2.30)

*Adjusted for age.

**Adjusted for age, marital status, smoking, drinking, physical activity, BMI, and community type.

## Discussion

The current study investigated the association between SES and MetS in Chinese adults, using the indicators of education, personal monthly income and household monthly income. Since gender differences have been observed in the relationship between socioeconomic position and CVD risk factors, we stratify the analyses by gender. The differences of SES on MetS between men and women were obvious. As for men, after multivariate adjustments, although the odds ratios for MetS increased with higher SES levels, the associations between them were not statistically significant. However, as for women, inverse associations were shown between SES and MetS. To be exact, the ORs decreased with higher SES levels. Lower SES was associated with a high risk for MetS among women but not in men. This was similar to other finding in Korea [5] and Portugal [19].

Generally, the prevalence of MetS varied between men and women too. It increased slightly with higher SES for men, while for women it showed an inverse association with SES. And the prevalence of each component of MetS among SES level was also different between men and women. These results were similar to those of Korean National Health and Nutrition Examination [5] in terms of overall MetS prevalence, abdominal obesity, high blood pressure and high blood glucose. However, as for high TG and low HDL, they presented obvious differences. For Chinese men, prevalence of high TG and low HDL increased with upgrading SES levels, while in Korea that of high TG and low HDL fluctuated a little among different SES levels. And for Korean women, the prevalence of high TG and low HDL decreased with increasing income, while that of high TG and low HDL showed no significant fluctuation for Chinese women. The reasons for the differences may lie in the fact that during the special economic transition period, Chinese men with higher SES had much more ability and opportunity to consume rich-in-fat foods, which can result in higher TG and lower HDL and Chinese women tend to be more concerned about their fitness. Studies found that income was positively associated with the consumption of snacks and excessive fried food and SES played a vital role in the early stage of eating behavior transition in China [20].

The possible mechanism for the impacts of SES on MetS may through individual’s unhealthy behaviors, such as smoking and drinking. Those with lower SES tended to be more likely to smoke and drink. In addition to that, smoking and drinking are both risk factors for cardiovascular diseases. This would further aggravate the potential risks to develop MetS.

Education has been found to be associated with MetS in many studies [5,10]. The significance that education is a strong predictor of health lies in that education may affect life-style behaviors, psychosocial attitude, accessibility to health services, and economically advantageous surroundings [5,21]. In China, education plays a special role for those with lower SES. It was instrumental to upgrade children belonging to the disadvantageous group to higher SES level, thus increasing their intergenerational income mobility [22].

The importance of income, income inequality, and their associations with health has been discussed in many literatures [23-25]. In our studies, we found significant association between personal monthly income or household monthly income and MetS after the multivariate adjustments for women but not for men. And income inequality was found to be associated with unequal access to health care and morbidity [26]. Apart from that, high income inequality increased the probability of health compromising behavior such as smoking and alcohol consumption [25], which were both risk factors for MetS.

Additionally, psychosocial stress may be considered as another factor caused by SES in the prevalence of MetS. Lower SES was usually associated with higher levels of cortisol [27], which was the risk factor for hypertension [28]. These findings might provide an alternative mechanism for the effects of SES on MetS. China is now in the great transition phase for economic and social structure. The polarization of SES, the rising expenditures for foods, education, medical care and housing prices may result in psychological stresses, especially among those with lower SES. This may further aggravate health inequality and diseases associated with MetS.

Apart from that, several factors may contribute to the gender differences in the association between SES and MetS. Women may experience a greater degree of discrimination in all spheres of life (e.g. labor market, pay, household work etc.), and women of low SES might have worse living conditions than low SES men [29]. Additionally, women of low SES are exposed to a range of stressors compared to either high SES women or low SES men.

The limitations of our study included the cross-sectional nature and a possible deficiency of causal inferences. In addition, as the information about SES was self-reported, some residents may not provide the precise income. However, the large sample of population represented for Beijing residents, and this enabled us to draw inferences of the association between SES and MetS among community residents in Beijing.

## Conclusions

In summary, lower SES was associated with higher risks of MetS among women Beijing residents but not for men. It is necessary to make greater efforts on part of the society, government, and institutions to reduce MetS inequality through management of education and income distribution. Apart from that, health education programs should be made specifically for men with higher SES in order to raise their awareness of MetS and reduce high TG and low HDL. Additionally, other intervention strategies could be proposed to help individuals quit smoking and drinking and cope with stress in both men and women with lower SES.

## Competing interests

The authors declare that they have no competing interests.

## Authors' contributions

JMY and DYH designed the study; YQZ, RQC, JLG, RJD, YYF, LJZ performed the data collection and health interviews; YQZ, JMY, RQC made the analysis and drafted the manuscript. All authors read and approved the final manuscript.

## Pre-publication history

The pre-publication history for this paper can be accessed here:

http://www.biomedcentral.com/1471-2458/12/921/prepub
